# Nontuberculous Mycobacteria and Laboratory Surveillance, Virginia, USA

**DOI:** 10.3201/eid3006.240431

**Published:** 2024-06

**Authors:** Isaac See, Kelly A. Jackson, Rebecca Byram, Nadege Charles Toney, Cheri Grigg, Shelley S. Magill

**Affiliations:** Centers for Disease Control and Prevention, Atlanta, Georgia, USA

**Keywords:** tuberculosis and other mycobacteria, nontuberculous mycobacteria, public health, Virginia, bacteria

**To the Editor:** We read with interest the recent article by Mullen et al. (*1*) that describes associations between *Mycobacterium avium* complex and *Mycobacterium abscessus* pulmonary disease prevalence and environmental exposures and risk factors. We applaud the innovation of efforts in states to better characterize nontuberculous mycobacteria (NTM) epidemiology.

In the discussion section of the article, the authors reference a publication describing results from a surveillance pilot for pulmonary and extrapulmonary NTM conducted by the Centers for Disease Control and Prevention (CDC) Emerging Infections Program’s Healthcare-Associated Infections–Community Interface Activity (*2*). The authors state, “Our study differed from that [CDC] study in multiple ways. Of note, we included data from a state in the southeastern United States, a region not represented in the CDC surveillance data, and gathered comprehensive surveillance data for the entire state from statewide laboratories rather than individual sentinel laboratories” (*1*). Although none of the sites in the CDC surveillance pilot conducted statewide surveillance for pulmonary NTM, we wish to clarify that the surveillance was active and also population-based (i.e., surveillance for isolation of NTM among all residents of certain counties) and was not a surveillance based on sentinel laboratories. Of note, in 2024 we have also recently expanded the geographic scope of Healthcare-Associated Infections–Community Interface Activity’s active, laboratory- and population-based NTM infection surveillance (including pulmonary NTM surveillance) to include Georgia as one of our surveillance sites. This addition will improve the surveillance program by adding representation from the southeastern United States.
